# Lyophilized equine platelet-rich plasma (L-GF^equina^) antagonize the Reproductive toxicity and oxidative stress Induced by Cyclophosphamide in female rats

**DOI:** 10.1186/s13048-023-01161-x

**Published:** 2023-04-28

**Authors:** Ahmed Sabry S. Abdoon, Ahmed M.E Al-Atrash, Seham S. Soliman, Amro M. El-Sanea, Amina A. Gamal el Din, Hossam M. Fahmy

**Affiliations:** 1 Department of Animal Reproduction and Artificial Insemination, Veterinary Research Institute, National Research Centre, Dokki, Cairo, 12622 Egypt; 2 Medical and Radio Protection Administration, Nuclear Materials Authority, Cairo, Egypt; 3 Department of Pathology, Medicine and Clinical Studies Research Institute, National Research Centre, Dokki, Cairo, 12622 Egypt; 4 Laboratory and Transfusion Medicine, Faculty of Medicine, Ain Shams University, Cairo, Egypt

**Keywords:** Rat, Reproductive toxicity, Ovary, Uterus, Blood profile, Antioxidants

## Abstract

**Background:**

The antineoplastic agent Cyclophosphamide (CP) induces reproductive toxicity. New strategies for protecting ovarian tissue damage in women with chemotherapy-induced reproductive toxicity are essential. This study was designed to evaluate the possible protective effect of combined treatment with L-GF^equina^ on CP-induced reproductive toxicity in the mature female rat.

**Methodology:**

Forty mature female rats were assigned into four groups: First group, control: rats were intraperitoneally injected (IP) with 200 µl sterile saline solution on days 1 and 10; Group 2 (CP): were IP injected with 75 mg/kg on days 1 and 10 to induce POI); Group 3 (CP + L-GF^equina^): as in group 2 + IP injected with 200 µl rehydrated L-GF^equina^ half-hour after CP injection on day 1 and 10); Group 4 (L-GF^equina^): rats were IP injected with 200 µl L-GF^equina^ on day 1 and 10). Blood samples were collected for a complete blood picture and determinations of nitric oxide and malondialdehyde. Animals were sacrificed on Day-21, and genitalia was dissected, weighed, and fixed in 10% formalin for histopathological and morphometric evaluation.

**Results:**

On day 21 of the experiment, body weight, ovarian parameters (Ovarian weight, uterine weight, the number of ovarian follicles, and corpora lutea (CL) were determined, and histopathological changes, blood profile, as well as antioxidant activity assessment, were performed. CP significantly suppresses ovarian and uterine functions and increased MAD, NO levels, RBCs, hemoglobin, WBCs, and platelet count compared to the control group ( P < 0.05). While, in CP + L-GF^equina^ group, gross, histomorphometry parameters, blood, and biochemical markers were similar to that in the control. IP injection of L-GF^equina^ alone significantly (P < 0.05) increased body weight, and ovarian and uterine morphometry compared with the control.

**Conclusion:**

co-administration of L-GF^equina^ with CP might protect the reproductive organs in rats through its high antioxidant capacity.

## Introduction

Cyclophosphamide (CP) is widely used as an antitumor and immunosuppressant drug for the treatment of breast cancer [1], prostate cancer [2] and as an immunosuppressive agent for the treatment of autoimmune and immune-mediated diseases [3]. After its oral administration, it is converted in the liver by the cytochrome P450 enzyme into phosphoramide mustard and acrolein [4]. The anticancer effects of CP are related to phosphoramide mustard, while its toxic side effects are related to acrolein [5]. Acrolein negatively affects biochemical reactions and creates oxidative stress in tissues. The reproductive toxicity of CP has been documented in human and experimental animals [6]. Previous studies have documented that the administration of CP caused a decrease in ovarian function and has detrimental effects on reproductive organs [7]. CP causes irreversible and progressive ovarian damage by follicular depletion, severe vascular damage, and the destruction of oocytes [8]. In mice treated with CP, dysfunction of the ovary is related to the destruction of the granulosa cells [9]. It acts by alkylation of the guanine compound of the DNA which results in crosslinking, miscoding, and DNA breakage [10]. In in vitro studies using human ovarian xenograft models, CP induces DNA breaks in primordial follicles, which trigger the apoptotic process and death in ovarian tissues [11]. In addition, few studies have explored the toxic effect of CP on uterine tissue, and CP induced uterine damage in rats [12].

Blocking any damage caused by the chemotherapy treatment would be the optimal way for maintaining reproductive function. In this respect, the administration of tyrosine kinase inhibitor imatinib [13], Sphingosine-1 Phosphate (SIP) [14], or gonadotropin-releasing hormone agonists (GnRHa) [15] have been used for the protection of ovarian function during treatment with chemotherapy. However, these procedures are not providing satisfactory recovery in chemotherapy-treated patients [16] and more research is needed to determine their usefulness, to ensure that they did not reduce the efficacy of chemotherapy, and to check their cytotoxicity. Therefore, new effective therapeutic strategies are necessary to manage reproductive toxicity in patients treated with chemotherapy.

Platelet-Rich Plasma (PRP) is a preparation of autologous plasma enriched with a platelet level above the baseline, and it plays an essential role in regenerative medicine. Platelets secrete growth factors and active metabolites that support the three phases of wound healing and tissue repair cascade (inflammation, proliferation, remodeling) [17]. In cyclophosphamide-induced ovarian failure rats, PRP treatment increased the ovarian cortex volume, pre-antral follicle number, and antral follicle diameter [18]. Also, histopathological studies revealed that PRP treatments improved both the quantity and the quality of follicles in mice ovaries compared to the control group, the PRP-treated group had a lower number of atretic follicles [19]. Most of the studies conducted on PRP use autologous or heterologous PRP from the same species, however, the lack of commercially available product, a standard method for the preparation of PRP, and variation among patients and their health conditions limits the use of PRP in clinical trials. Recently, intrauterine infusion of equine lyophilized platelet-rich plasma (L-GF^equina^) increased the endometrial thickness, and pregnancy rate and improve fertility in repeat-breeding purebred Arabian mares [20], and L-GF^equina^ increased the number of recovered oocytes and blastocysts production after ovum-pick up in vitro embryo production in Holstein cows [21]. There is no available literature on the efficacy of lyophilized or PRP from other species in restoring reproductive function in CP-treated animals. Therefore, this study was designed to investigate the possible protective effects of intraperitoneal injection of L-GF^equina^ shortly after CP injection on reproductive tract weight, follicular development, ovarian and uterine histology and morphometry, blood profile, and antioxidant capacity in rats treated with CP.

## Materials and methods

### Animals & ethics

Forty healthy mature female Wistar Albino rats (aged 3–4 months) obtained from the Egyptian Holding Company for Biological Products and Vaccines (VACSERA, Egypt) were used in this work. Animals were housed in sterilized polypropylene rats’ cages under a 12/12-h light/dark cycle at an ambient temperature of 25 °C. Food and water were provided *ad libitum*. The experimental protocol for the in vivo experiments was approved by the Institutional Animal Care and Use Committee of the National Research Centre of Egypt (Approval no: RO120201).

### Lyophilized L-GF^equina^ (L-GF^equina^)

L-GF^equina^ consists of lyophilized horse platelet-rich plasma. It is produced by a patented method developed by Dr. Hossam M. Fahmy **(Eu Patent NO. 16199628.5–1466)** [22]. The activation of platelets in vitro before lyophilization led to a release of supra-physiological doses of growth factors. Among these are transforming growth factor-β, platelet-derived growth factor, insulin-like growth factor, and fibroblastic growth factor. Each vial contained platelet-derived growth factors equivalent to those found in platelets derived from 20 mL of whole blood, and platelet counts 3–5 times that of the normal baseline. To reconstitute the solution, 10 ml of sterile saline solution was added to a bottle of L-GF^equina^.

### Experimental design

**Fig. 1:**
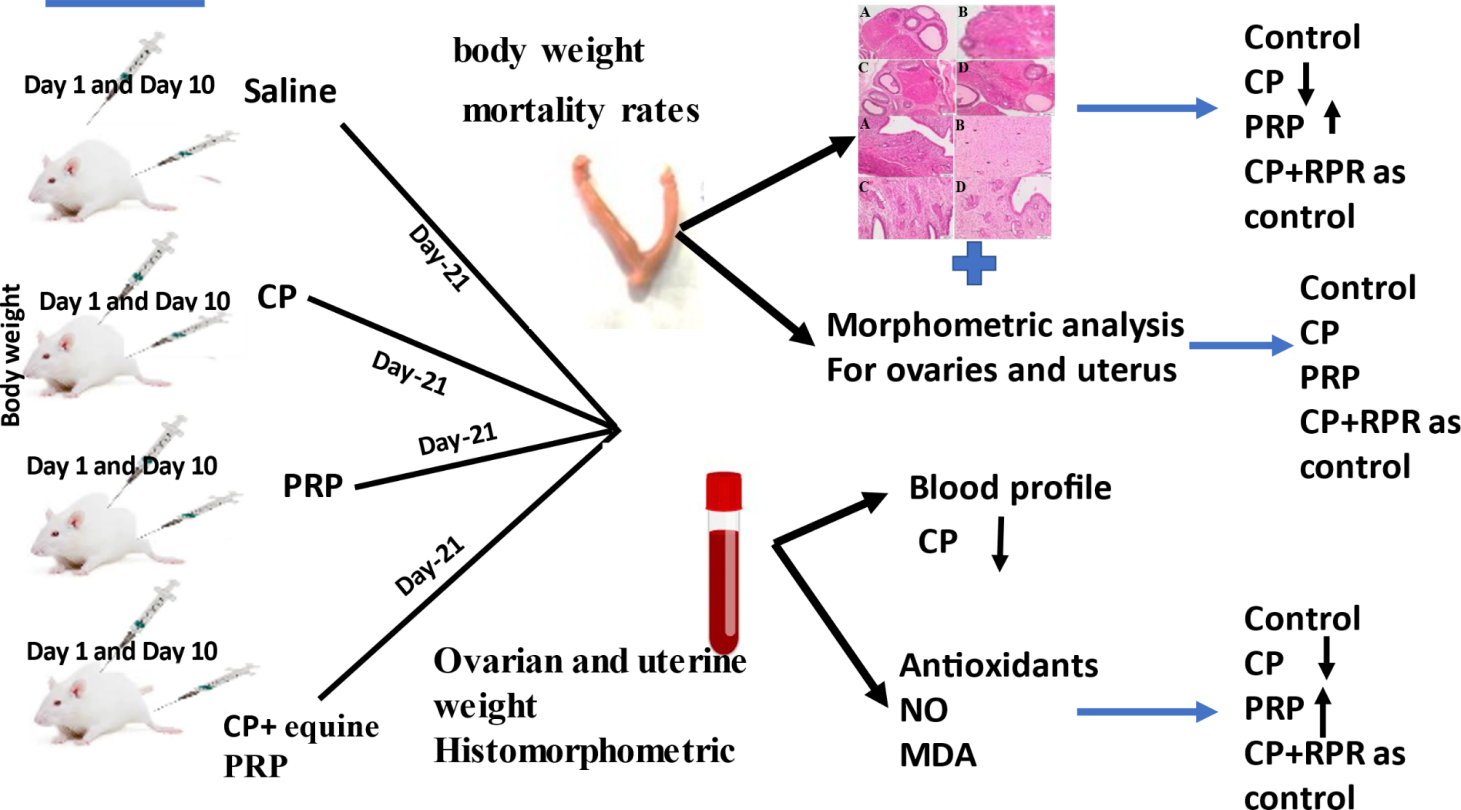
Schematic representation of the experimental design used for the cyclophosphamide (CP) -induced premature ovarian insufficiency and treatment by equine platelet-rich plasma Equine PRP)

The dose of the cyclophosphamide used in this study was determined according to Ahmadian et al. [19]. Cyclophosphamide is inactivated after one week in rats, so two successive doses with week intervals were used in the present study according to Angley et al. [23]. After two weeks of adaptation, body weight was determined, and experimental Rats were randomly divided into four groups to examine the protective effect of L-GF^equina^ against the gonadotoxicity of cyclophosphamide (n = 10 in each group). Group 1 (Control): female rats were intraperitoneally (IP) injected with 200 µl saline solution on Days 1 and 10 and animals were sacrificed on Day-21. Group 2 (Cyclophosphamide (CP), Endoxan, Baxter Oncology GmbH, Kantstrasse 2, Germany): where rats were IP injected with 75 mg/kg on Day-1 and 10 to induce POI). Group 3(CP + L-GF^equina^), rats were IP injected with 200 µl rehydrated L-GF^equina^ half an hour after CP injection on Day-1 and 10. Group 4 (L-GF^equina^): rats were IP injected with 200 µl rehydrated L-GF^equina^ on Days 1 and 10. At the end of the experiment, body weight was determined. Also, the mortality rate was recorded during the study (Fig. 1).

#### Sample preparation

During the study, the mortality rate was recorded and at the end of the experiment on day-21, all the rats were weighed and anesthetized by an intramuscular injection of 50 mg/kg ketamine hydrochloric acid (Ketam; EIPICO, 10th of Ramadan City, 1st Industrial Zone B1, Egypt) and 7 mg/kg xylazine hydrochloric acid (Xyla-Ject, Adwia, 5ht Settlement, New Cairo, Cairo, Egypt), blood samples were collected from the left ventricle using a syringe, left for 10 min to clot, and centrifuged at 3000 rpm for serum separation. The separated serum was stored at -80 °C for further determinations of antioxidant capacity (nitric oxide and malondialdehyde). Rats were sacrificed by cervical dislocation and a ventral midline incision was performed to expose the reproductive organs then genital tract and ovaries were dissected, and ovaries were checked for the number of ovarian follicles and corpus luteum under a stereomicroscope (Zeiss, Germany), and ovarian and uterine weight was recorded in grams with a precision of 0.0000 using a digital balance (Radwag, Torunska 5, Poland).

#### Histopathological examination and ovarian and uterine morphometry

For light microscopic evaluation, ovarian tissues were fixed in 10% formalin and embedded in paraffin wax and cut into five µm thick sections, mounted on slides, and stained with hematoxylin and eosin (H&E) for routine histopathologic study [24]. The sections were examined using an Olympus CX41 research microscope. Histopathological examination of the tissue damage was performed regarding each parameter, such as inflammatory cells, vascular congestion, epithelial degeneration, and vacuoles in epithelial cells and dilated endometrial gland.

The morphometric analyses were performed using the Leica Qwin 500 Image analyzer (LEICA Imaging Systems Ltd, Cambridge, UK), which consisted of a Leica DM-LB microscope with a JVC colour video camera attached to a computer system. Morphometric parameters included in this study were the area % of endometrial glands and stroma, and the count of different types of ovarian follicles. The morphometric analysis was carried out on hematoxylin and eosin-stained slides [25]. The nuclear area was measured at magnification×100. The selected nuclei were surrounded by a line to be covered automatically by a green mask, which is called a binary image. The parameters of these binary images appeared automatically in the form of a table in micrometers, and finally, the total number of all parameters examined was determined. All morphometric evaluations were conducted by the same investigator who was blind to the clinical data at the time of examination.

#### Blood profile

Blood samples with anti-coagulant EDTA were analyzed for hematological parameters of red blood cell (RBCs) counts, White Blood Cell (WBC) counts, and the total number of lymphocytes, granulocytes, and platelet counts using an automated blood cell counter (COUNTENDER20+, SFRI, Bordeaux, France).

#### Biochemical analysis

Serum activities of Malondialdehyde (MDA) and Nitric oxide (NO) were determined calorimetrically using kits (Biodiagnostic, Giza, Egypt) according to the methods of Ohkawa et al. [26] and Montgomery and Dymock [27], for MDA and NO, respectively.

### Statistical data analyses

Results were expressed as the mean ± SE. The significant differences between groups were determined using One-way ANOVA with LSD post hoc tests, followed by the Duncan test in all cases. All samples were tested for normality according to the Shapiro-Wilk test before ANOVA. P values < 0.05 were considered statistically significant. Different letters indicate statistically significant differences between groups, which means that sharing the same letter does not differ significantly. Data were analyzed by using SPSS Statistics for Windows, Version 20 (IBM SPSS Statistics for Windows, Version 20. Armonk, NY: IBM Corp).

## Results

### Body weight and mortality rate in control and experimental rats

As presented in Table 1, at the end of the experiment, results revealed that IP injection of CP significantly (P < 0.05) decreased body weight and increased (P < 0.05) mortality rate than in control or CP + L-GF^equina^ groups. Meanwhile, IP injection of L-GF^equina^ alone significantly (P < 0.05) increased body weight more than control or CP + L-GF^equina^ groups.

**Table 1:** Effect of CP, L-GF^equina^ or CP + L-GF^equina^ on body weights and mortality rate in rats (Mean ± SE)

Groups	Body Weight before (g)	Body weight after (g)	Mortality rate (%)
Control	156.6 ± 1.2^a^	175 0.0 ± 6.0^b^	0.0^b^
CP	159.6 ± 1.7^a^	146.2 ± 6.2^c^	30.0^a^
L-GF^equina^	158.9^a^ ± 1.4^a^	194.3 ± 4.8^a^	0.0^b^
CP + L-GF^equina^	154.8 ± 1.1^a^	176.7b ± 3.6^b^	10.0^b^

a,b within the same column differ significantly at P < 0.05

b,c within the same column differ significantly at P < 0.05

### Effect of treatment on reproductive organs weight

**Fig. 2:**
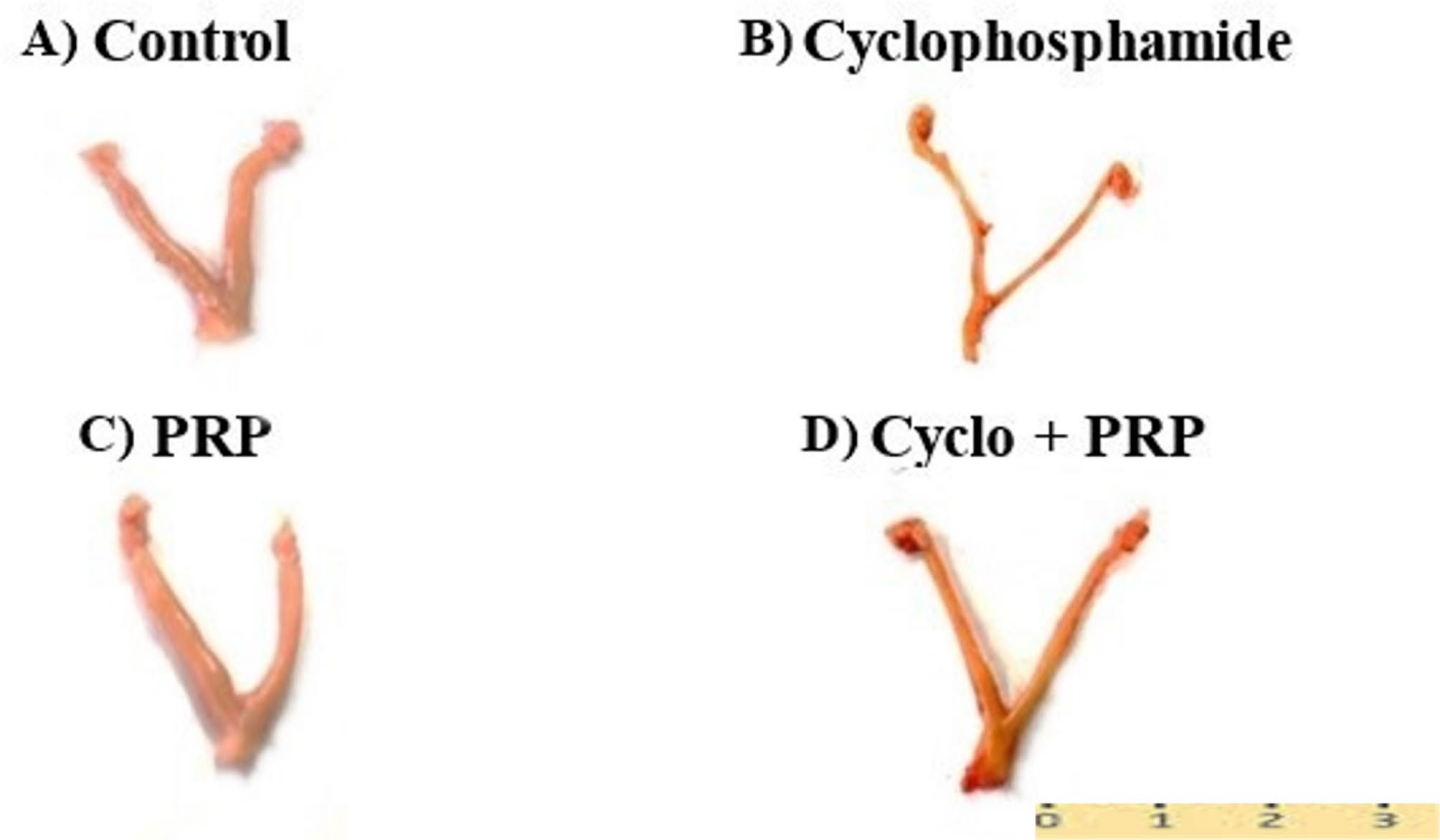
Photographs showing genital tract dissected from control (**A**), cyclophosphamide (**B**), Equine PRP (**C**) and Cyclophosphamide + Equine PRP groups

Data illustrated in Table 2; Fig. 2 represent the effect of treatment with CP with or without L-GF^equina^ on reproductive organ weight in rats. Results revealed that there was a significant (P < 0.05) decrease in the reproductive tract, left ovary, and uterine weights in the CP group (Fig. 2B) compared with the control animals (Fig. 2A). In rats injected with PRP alone, reproductive tract weight, and uterine weight were higher (P < 0.05) than in the other groups (Fig. 2C)). While, no significant difference was detected for the reproductive tract, ovarian and uterine weights for the control and CP + L-GF^equina^ group (Fig. 2D).

**Table 2:** Effect of IP injection of CP, L-GF^equina^ or CP + L-GF^equina^ on ovarian and uterine weight in rats (Mean ± SE)

Groups	Reproductive tract weight (g)	Ovarian weight (g)		Uterus weight (g)
		Right	Left	
Control	0.42 ± 0.04^b^	0.05 ± 0.01^a^	0.05 ± 0.005 ^a^	0.3 ± 0.03^a,b^
CP	0.27 ± 0.01 ^c^	0.04 ± 0.002 ^a^	0.03 ± .003^b^	0.2 ± 0.01 ^c^
L-GF^equina^	0.60 ± 0.01 ^a^	0.05 ± 0.004 ^a^	0.05 ± 0.005 ^a^	0.5 ± 0.01 ^a^
CP + L-GF^equina^	0.40 ± 0.02 ^b^	0.05 ± 0.003 ^a^	0.04 ± 0.003 ^a^	0.3 ± 0.02 ^a,b^

a,b within the same column differ significantly at P < 0.05

b,c within the same column differ significantly at P < 0.05

### Histopathological changes

**Fig. 3:**
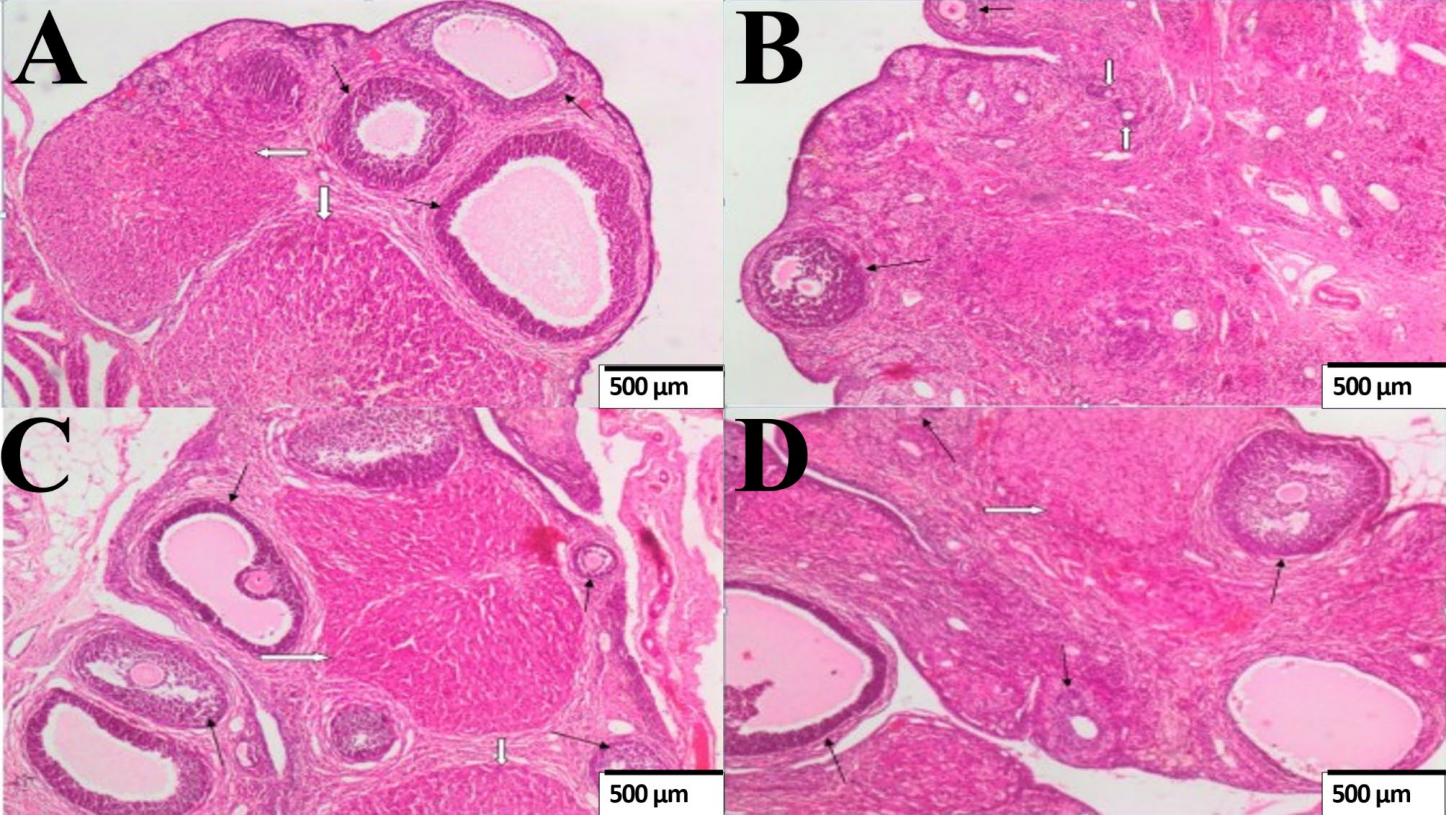
Ovarian section of the control rats (**A**) showing antral follicles (Black arrows), and corpora lutea (White arrows); **B**) Ovarian sections of CP treated group showing degenerated ovarian tissue, depletion of follicles, wide areas of stroma, and scattered degenerated follicles (White arrows), next to the secondary follicle (Black arrow). **C**) Ovarian sections of rats treated with Equine PRP showing several follicles at variable stages of development and maturation (Black arrows) and corpora lutea (White arrows); **D**) Ovarian section of rats treated with CP + Equine PRP showing several follicles at variable stages of development and maturation (Black arrows) and corpora lutea (White arrow) (H&E x40, bar 500 nm)

Ovarian tissue in control rats showed normal architecture, and multiple follicles was seen at various stages of development and maturation. These included primordial, primary, and secondary follicles and multiple mature Graafian follicles. Corpora lutea were also observed formed of sheets of lutein cells with ample acidophilic cytoplasm and basophilic nuclei (Fig. 3A). Ovarian tissue of rats treated with CP showed degenerated tissues with depleted follicles; primordial, primary, and mature follicles compared to the control animals (Fig. 3B). Graafian follicles showed separation of cells and loss of the cumulus oophorous, shrunken granulosa cells, and several atretic follicles were observed. Ovarian tissue in rats treated with L-GF^equina^ showed normal architecture with multiple follicles at various stages of development and maturation, together with multiple corpora lutea (Fig. 3C). Ovarian tissue of CP + L-GF^equina^ showed ovarian architecture similar to control. Multiple follicles at all stages of development and maturation, corpora lutea occupied the ovarian cortex and no atretic follicles were detected (Fig. 3D).

**Fig. 4:**
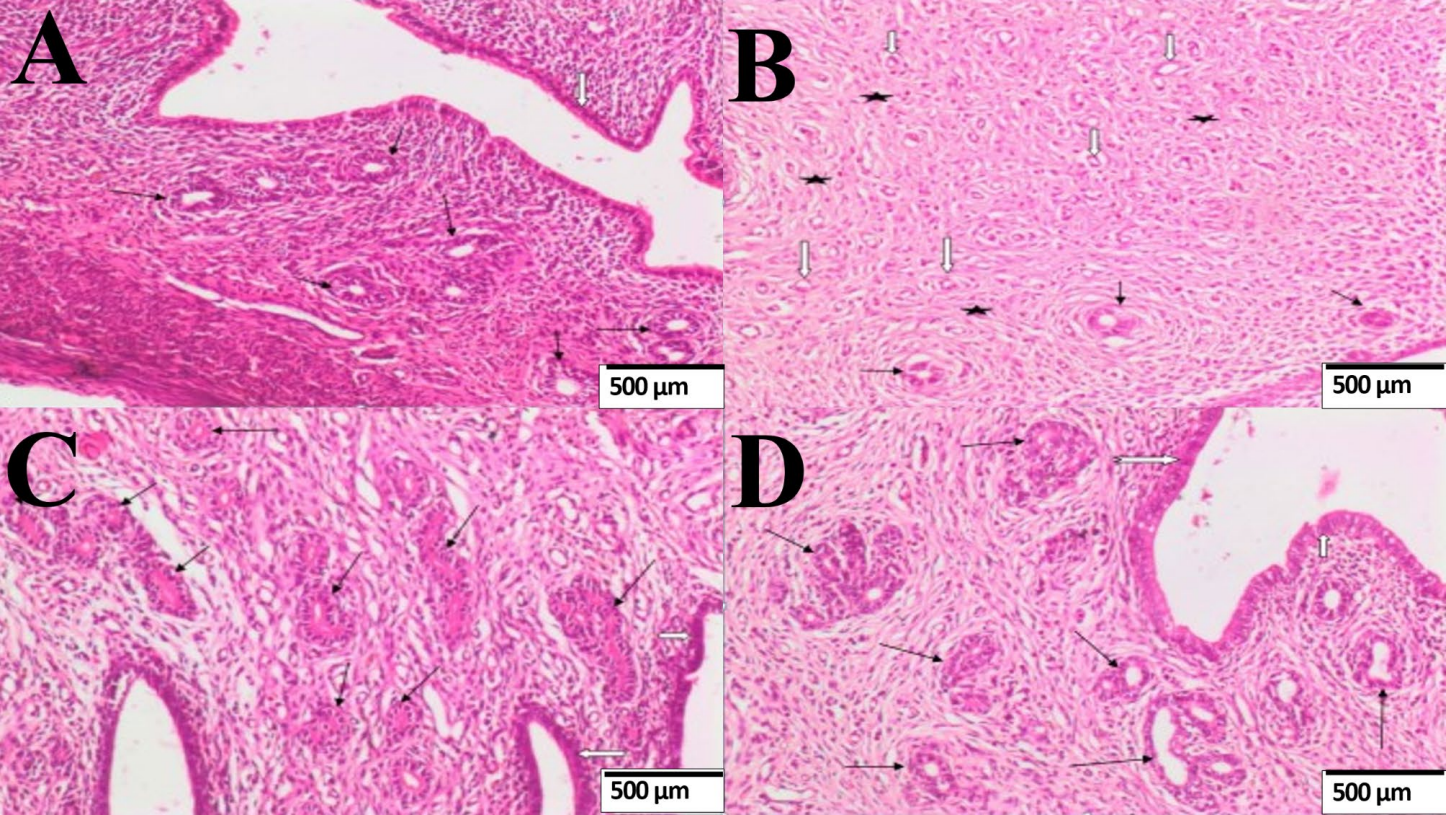
Uterine tissue sections of a control rat (**A**) showing several proliferating endometrial glands (Black arrows) surrounded by stroma, together with intact non degenerated endometrial lining (White arrow); **B**) Uterine tissue sections of rat treated with CP showing decreased inactive endometrial glands (Black arrows), fibrotic stroma (Black stars) and numerous blood vessels (White arrows); **C**) Uterine tissue sections of rat treated with Equine PRP showing several proliferating endometrial glands (Black arrows) and intact non degenerated high columnar epithelial endometrial lining (White arrows); **D**) Uterine tissue sections of rats treated with CP + Equine PRP showing numerous proliferating endometrial glands (Black arrows) with intact non degenerated endometrial lining (White arrows) and decreased stromal fibrosis(H&E x100, bar 500 μm)

Regarding the effect of IP injection of CP, CP + L-GF^equina^, or L-GF^equina^ on histology of the uterus in rats: The control and the L-GF^equina^ treated groups showed normal endometrium with numerous, proliferating endometrial glands exhibiting non-dilated lumina. Lining epithelium consisted of active, high columnar epithelium. Stroma was an active form of proliferating spindle cells (Fig. 4A). In CP-treated animals, histopathological changes were noticed in the uterus such as increased epithelial degeneration, inactive low cuboidal to the flattened epithelial lining, vacuoles within the epithelial lining, and focally dilated endometrial glands, and densely fibrotic stroma. Decreased glandular proliferation was observed, and vascular proliferation and numerous blood vessels were also seen (Fig. 4B). The uterus of the L-GF^equina^ treated groups showed active endometrium with numerous, proliferating endometrial glands exhibiting non-dilated lumina. The lining epithelium was active and consisted of high columnar cells. Stroma was an active form of proliferating spindle cells (Fig. 4C). The groups treated with CP + L-GF^equina^ showed signs of fibrosis, increased cellular proliferation of the endometrium, decreased vacuoles within the epithelial lining, and a decrease in the vascular proliferation compared to the CP-treated group (Fig. 4D).

### Effect of treatment on ovarian morphology

Table 3 represents the effect of the treatment of rats with CP, L-GF^equina^ or CP + L-GF^equina^ on ovarian morphology. In CP-treated rats, the number of follicles and CL in both the right and left ovary and the total number of follicles and CL per ovary were significantly (P < 0.05) lower than in the other groups. Meanwhile, in L-GF^equina^ injected rats, the number of ovarian follicles and CL was significantly (P < 0.05) higher than in control or CP + L-GF^equina^. In CP + L-GF^equina^-treated rats, the number of ovarian follicles and CL was similar to that in the control group.

**Table 3:** Effect of CP, L-GF^equina^ or CP + L-GF^equina^ on follicles and CL number (Mean ± SE)

Groups	No follicles/ovary		Total no follicles /animal	No CL/ovary		Total no CL/animal
	Right	Left		Right	Left	
Control	3.2 ± 0.8^a,b^	1.6 ± 0.4^a,b^	4.8 ± 0.6^a,b^	4.0 ± 0.5^a,b^	4.9 ± 0.7^a,b^	8.6 ± 0.6^a,b^
CP	0.0^c^	0.4 ± 0.2^c^	0.4 ± 0.1^c^	1.1 ± 0.3^c^	0.0^c^	1.1 ± 0.2^c^
L-GF^equina^	5.9 ± 0.6^a^	3 ± 0.3^a^	8.9 ± 0.4^a^	7.7 ± 0.9^a^	7.2 ± 0.9^a^	14.9 ± 0.9^a^
CP + L-GF^equina^	3.0 ± 0.3^a,b^	1.7 ± 0.3^a,b^	4.7 ± 0.3^a,b^	5.2 ± 0.5^a,b^	5.6 ± 0.6^a,b^	10.8 ± 0.5^a,b^

a,b within the same column differ significantly at P < 0.05

b,c within the same column differ significantly at P < 0.05

### Ovarian and uterine morphometric analysis

The effect of treatment with CP, L-GF^equina^ or CP + L-GF^equina^ on ovarian and uterine morphometric measures is presented in Table 4. Results showed that treatment with CP significantly (P < 0.05) decrease the number of primordial, primary, secondary, and antral follicles, and increased (P < 0.05) the number of atretic follicles compared with the control group. A significantly (P < 0.05) higher number of primordial, primary, secondary, and antral follicles and CL was recorded in the L-GF^equina^-treated group than in the other groups. In CP + L-GF^equina^-treated rats the number of primordial, primary, secondary, and antral follicles was similar to the control group.

**Table 4:** Changes in number of primordial, primary, secondary, antral, atretic follicles and CL in ovaries of CP, L-GF^equina^ and CP + L-GF^equina^ treated rats

Group	Primordial fol.	Primary fol.	Secondary fol.	Antral fol.	CL	Atretic foll
Control	34^a,b^	33^a,b^	12^a^	4^b^	1^b^	4^c^
CP	23^c^	19^c^	9^b^	2^b^	1^b^	37^a^
L-GF^equina^	55^a^	40^a^	15^a^	13^a^	3^a^	15^a,b^
CP + L-GF^equina^	30^a,b^	29^a,b^	13^a^	3^b^	2^b^	5^c^

a,b within the same column differ significantly at P < 0.05

b,c within the same column differ significantly at P < 0.05

Regarding the uterine tissue samples, the present work revealed that IP injection of CP in female rats produced a significant (P < 0.05) decrease in the glandular area and an increase (P < 0.05) in the stroma and dilated glands area compared to the control rats. In the meantime, IP injection of L-GF^equina^ significantly (P < 0.05) increased the glandular area and dilated glands compared with the control or CP + L-GF^equina^ groups. Meanwhile, the glandular and stromal areas were similar between the CP + L-GF^equina^ and the control group (Table 5).

**Table 5:** Morphometric changes in uterus of control, CP, L-GF^equina^ and CP + L-GF^equina^ treated rats (Mean ± SE)

Group	Glands	Stroma	Dilated glands
Control	2.184 ± 0.133^b^	18.701 ± 1.121^a^	1.825 ± 0.053^c^
CP	0.858 ± 0.121^c^	45.439 ± 1.866^b^	8.097 ± 1.929^a^
L-GF^equina^	6.488 ± 0.205^a^	18.446 ± 1.567^a^	4.643 ± 0.235^b^
CP + L-GF^equina^	2.327 ± 0.169^b^	16.938 ± 0.798^a^	3.051 ± 0.357^b^

a,b within the same column differ significantly at P < 0.05

b,c within the same column differ significantly at P < 0.05

### Blood profile

Data demonstrated that IP injection of CP significantly (P < 0.05) decreased RBCs, HB, hematocrit, WBCs, lymphocytes number, percentage, granulocytes number and percentage, and platelet distribution width. While CP treatment significantly (P < 0.05) increased procalcitonin, platelet distribution width, and platelet large cell ratio compared with the other groups. In CP + L-GF^equina^ and L-GF^equina^ treated groups, RBCs, HB, and hematocrit values were significantly (P < 0.05) higher than in other groups. IP injection of CP + L-GF^equina^ significantly (P < 0.05) increased platelet distribution width and red cells distribution width compared with the other groups (Table 6).

**Table 6:** Effect of treatment with CP, L-GF^equina^ or CP + L-GF^equina^ on blood parameters in female rats (Mean ± SE).

Item Group	Control	CP	Equine PRP	CP + L-GF^equina^
Red blood cell count (10^6^/µl)	6.4 ± 0.1^a^	5.6 ± 0.4^b^	6.7 ± 0.2^a^	6.9 ± 0.2^a^
Hemoglobin (g/dl)	13.8 ± 0.3^b^	12.1 ± 0.5^b^	15.3 ± 0.6^a^	15.1 ± 0.5^a^
Hematocrit (%)	38.8 ± 0.9^b^	36.4 ± 1.9^b^	46.3 ± 1.4^a^	46.3 ± 1.4^a^
MCV (fL)	60.8 ± 0.8^b^	64.4 ± 1.5^a^	65.1 ± 0.4^a^	65.1 ± 0.4^a^
Mean Corpuscular hemoglobin	21.9 ± 0.3	21.7 ± 0.5	22.6 ± 0.1	22.6 ± 0.1
MCHC (pg)	34.9 ± 1.5	34.2 ± 0.8	36.3 ± 0.4	35.1 ± 0.3
Procalcitonin (µg/L)	0.3 ± 0.03^c^	0.9 ± 0.01^a^	0.2 ± 0.03^c^	0.5 ± 0.04^b^
White blood cell count (10^3^/µl)	4.9 ± 0. 4^a^	3.7 ± 0.3^b^	5.9 ± 0.4^a^	5.5 ± 0.1^a^
Lymphocyte percentage (%)	74.8 ± 0.9^a^	68.1 ± 1.8^b^	76.4 ± 2.0^a^	75.5 ± 1.6^a^
Lymphocyte number(10^3^/µl)	3.5 ± 0.6^a^	2.7 ± 0.4^b^	3.7^a^ ± 0.1^a^	3.6 ± 0.3^a^
Granulocyte number (10^3^/µl)	0.5 ± 0.1^a^	0.2 ± 0.1^b^	0.5 ± 0.1^a^	0.4 ± 0.03^a^
Granulocyte percentage (%)	12.9 ± 0.6^a^	9.6 ± 1.1^b^	12.4 ± 0.7^a^	13.2 ± 0.4^a^
Platelet large cell ratio (%)	7.0 ± 0.2^a^	10.6 ± 0.4^b^	6.7 ± 0.4^a^	7.1 ± 0.4^a^
Platelet count (fL)	456.5 ± 42.9^b^	159.4 ± 8.2^c^	340.4 ± 19.3^c^	611.6 ± 46.1^a^

a,b within the same row differ significantly at P < 0.05

b,c within the same row differ significantly at P < 0.05

### Antioxidant capacity

**Fig. 5:**
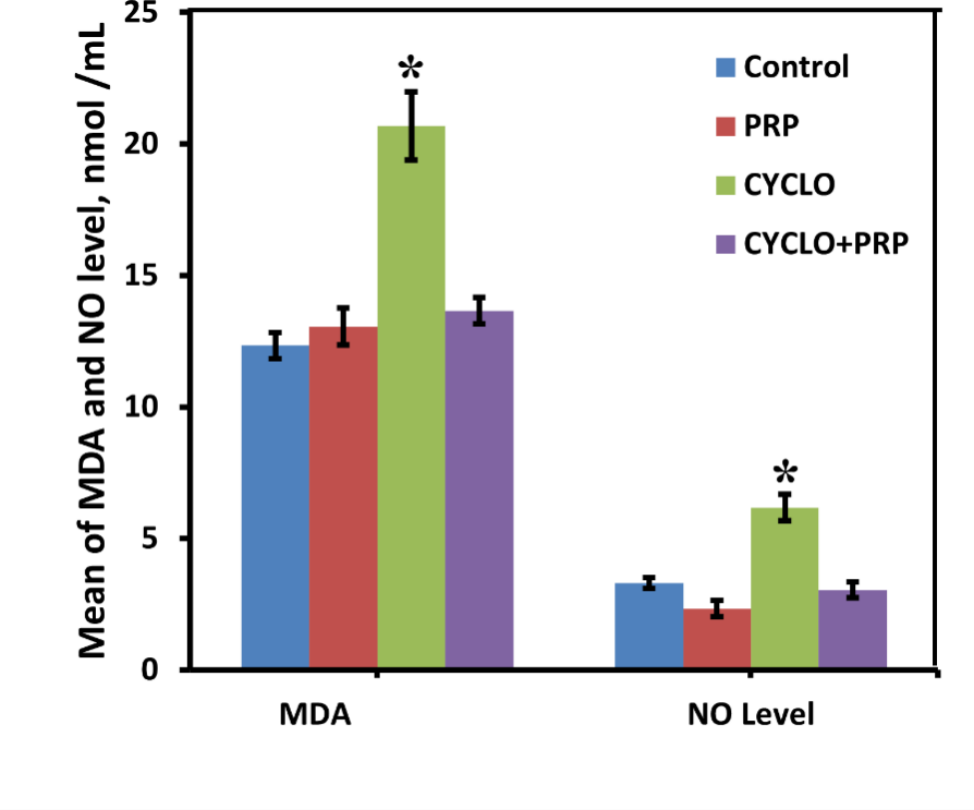
Histogram showing malonaldehyde and nitric oxide levels in control, CP, CP + Equine PRP and Equine PRP treated rats

Results showed a significant (P < 0.05) increase in serum nitric oxide and MDA levels among CP-treated groups in comparison with the other groups. There was no significant difference between the control and CP + L-GF^equina^ or L-GF^equina^ groups in terms of MDA and NO levels (Fig. 5).

## Discussion

In women, ovarian inactivity and infertility are the major problems associated with chemotherapy treatment. There is strong evidence that alkylating agents such as cyclophosphamide are strongly gonadotoxic, it produces ovarian failure in 42% of women treated [28]. The effect is occurring immediately during treatment and induces amenorrhea resulting from the loss of the growing follicles population [8]. Therefore, the preservation of fertility in women treated with chemotherapy is a priority of great importance. The high concentration of hormones, macrophages, neutrophils, cytokines, and various growth factors in PRP might contribute to tissue healing, regeneration, anabolism increase, differentiation, proliferation, angiogenesis activation, and inflammation control [29]. The use of PRP is considered a potentially successful opportunity to increase the fertility outcome in patients where the main problem is gonadotoxicity. However, in most of the studies conducted, autologous or heterologous PRP was used. This work aimed to investigate the possible protective role of lyophilized equine platelet-rich plasma (L-GF^equina^) on reproductive function in CP-treated mature female rats.

In the present work, in CP-treated rats, a significant decrease in body weight and an increase in mortality rate was recorded compared with the other groups. This decrease in body weight could be due to the direct effect of CP on different body organs and systems [30]. Meanwhile, the group injected with CP + L-GF^equina^ showed a body weight and mortality rate similar to that of the control group. So, IP injection of L-GF^equina^ shortly after CP might protect the body weight and systems from the adverse effect of CP. In addition, CP-treated rats showed a significant decrease in the uterus, and ovarian weight and a reduced number of ovarian follicles and CL. Histopathological examination of ovaries and uterus of CP-treated rats showed degenerative changes including depleted primordial, primary, and mature follicles with oedema and separation of cells, and loss of the cumulus oophorous. Granulosa cells were shrunken, and several atretic follicles were observed. In the uterus, epithelial degeneration, vacuoles within the epithelial lining, decreased glandular proliferation, and increased stromal fibrous tissue. Similar results were previously reported in rats [18, 31] and mice [32]. This side effect of CP is linked to follicle atresia and granulosa cell apoptosis [33]. Also, CP has a damaging effect on the ovarian stroma and vasculature [34], and damage to the granulosa cells will result in indirect damage to the oocytes, leading to germ cell loss [28]. CP diminished primordial follicles via follicle burnout [35], and induction of DNA damage and/or oxidative stress that triggers the activation of apoptosis in primordial follicles [36]. Furthermore, the present results indicated that treatment L-GF^equina^ shortly after CP injection could neutralize the adverse effects of CP on the ovaries, uterus, blood profile, and antioxidant activity. In the CP + L-GF^equina^ group, ovarian and uterine weights, the number of ovarian follicles, and CL were close to that in the control group. Histopathological examination of ovarian and uterine tissues showed similar architecture to that of the control animals. Numerous follicles at all stages of development and maturation are comparable to that in the control animals. Corpora lutea were also observed, and no ovarian and uterine stromal fibrosis was detected. These results are consistent with previous studies in which PRP administrated into the ovaries might protect and/or restore ovarian function in Cy-induced POI [19, 37]. In women, the intraovarian PRP injection resulted in a progressive increase in E2 and AMH levels and decreased the levels of FSH and LH [38]. Besides the local effect of PRP on ovarian function, PRP might positively affect the Hypothalamus Pituitary Ovarian axis (HPO axis). The high concentration of The TGF-β in PRP could affect the expression of the FSH receptor (FSHR), thus this interaction (between FSH-FSHr) induces survival stimuli for the antral follicles [39]. Also, TGF-β might increase the expression of the LH receptor (LHR), where LH and progesterone can inhibit follicular apoptosis. In addition, TGF-β, IGF, VEGF, and FGF, might have a significant role in the regulation of follicular growth and maturation [39]. The mechanism of action of L-GF^equina^ on the ovary is not entirely clear. The protective effect of L-GF^equina^ against the reproductive toxicity induced by CP might be due to the high concentration of growth factors in L-GF^equina^ that inhibit cytokines release, decrease inflammation, and result in tissue protection. L-GF^equina^ could also establish a balance between apoptosis and cell survival due to the presence of pro-apoptotic factors and anti-apoptotic factors [40], as indicated by the close antioxidant activity of NO and MDA in the CP + L-GF^equina^ group when compared to the control ones.

Moreover, in this work, rats that were treated with CP showed a significant decrease of RBCs, hemoglobin, hematocrit, WBCs, lymphocytes, and granulocytes values, and the oxidative stress markers MDA and NO values than control animals. While procalcitonin was higher in the CP group than control ones. These data are consistent with previous results [41, 42]. CP has side effects on the hematopoietic system leading to anemia, neutropenia, thrombocytopenia, secondary tumors, and genital abnormalities [43]. CP can prevent the proliferation of hematopoietic and immune cells in bone marrow [44]. Also, CP leads to oxidative stress through the strong depletion of antioxidants enzyme activities and the high production of prooxidants molecules [45]. The direct effect of the administration of PRP on blood profile in humans or animals has never been examined before. In this work, simultaneous treatment of rats with the L-GF^equina^ at the time of CP treatment protect the hematopoietic system from the adverse effects of CP on blood cell profile and keeps the oxidative stress parameters at normal levels. Interestingly, the administration of L-GF^equina^ at the time of CP treatment significantly increases the platelet count compared with CP or control groups. Therefore, one of the mechanisms by which L-GF^equina^ exerts its action might be by stimulating the bone marrow to produce more blood cells via its higher content of vascular endothelial growth factor (VEGF).

In addition, in the present work, there was a significant increase in body weight in rats injected with L-GF^equina^ alone compared with the other groups. Also, IP injection of L-GF^equina^ significantly increased reproductive uterine weight, and it also significantly increased the number of ovarian follicles and CL compared with the other groups. The ovarian tissue of rats treated with L-GF^equina^ showed normal architecture with a significantly higher number of primordial, primary, secondary, and antral follicles compared with the other groups. Also, L-GF^equina^ significantly (P < 0.05) increase endometrial glands and their secretions compared with the control group. The high concentration of cellular signals contained in PRP might act by favoring the recruitment of latent follicles simply by stimulating them with numerous growth factors [46]. Therefore, the high concentration of growth factors present in L-GF^equina^ might be able to stimulate ovarian stem cells and induce their differentiation in ex-novo oocytes.

In conclusion, lyophilized PRP from other species such as L-GF^equina^ might protect the reproductive function and body systems at a normal level in CP-treated rats through its high antioxidant capacity that protects the body organs and systems from the damage produced by CP.

## Data Availability

The datasets generated during and/or analyzed during the current study are available from the corresponding author upon reasonable request.
